# Task‐Evoked Functional Activation and Coupling With CSF Flow Detected in the Human Brain With Ultrashort Echo Time fMRI at 7 T

**DOI:** 10.1002/nbm.70342

**Published:** 2026-06-21

**Authors:** Sara Ponticorvo, Ekaterina Paasonen, Pavel Filip, Douglas L. Rothman, Juha S. Valjakka, Olli Gröhn, Shalom Michaeli, Silvia Mangia

**Affiliations:** ^1^ Department of Radiology, Center for Magnetic Resonance Research University of Minnesota Minneapolis Minnesota USA; ^2^ A. I. Virtanen Institute for Molecular Sciences University of Eastern Finland Kuopio Finland; ^3^ Neurocenter Kuopio University Hospital Kuopio Finland; ^4^ General University Hospital, Charles University Prague Czech Republic; ^5^ Department of Radiology and Biomedical Imaging, Magnetic Resonance Research Center Yale University New Haven Connecticut USA

**Keywords:** CSF, fMRI, glymphatic system, high‐field, inflow, neurofluids, ultrashort echo time, zero echo time

## Abstract

Ultrashort echo time (UTE) and zero echo time (ZTE) techniques have been shown to generate robust functional MRI (fMRI) contrast of hemodynamic origin from the inflow of unperturbed spins into the imaging volume under local radiofrequency transmission. As such, they are ideally suited not only to map the dynamics of cerebral blood flow (CBF) during intense neuronal activity but also the dynamics of cerebrospinal fluid (CSF). The goal of this work was to pilot the use of UTE‐fMRI for human studies of brain activation and neurofluid dynamics. We thus conducted fMRI studies at 7 T with a transmit/receive head coil using a slab‐selective UTE sequence on 13 human participants during a visual task. Spatio‐temporally matched gradient‐echo echo planar imaging (GE‐EPI) data were also acquired for qualitative comparison purposes, and respiratory and cardiac signals were measured to quantify multiple physiological metrics. Functional brain activations were analyzed using a general linear model at single subject and group levels. Moreover, time‐courses from the whole cortex, carotid arteries, and fourth ventricle were correlated between each other, and physiological contributions to these ROI signals were evaluated with a linear mixed model and a relative importance computation. Robust and reproducible functional activations were detected with UTE‐fMRI in the visual cortex across participants. Although the functional contrast‐to‐noise ratio was higher with GE‐EPI than with UTE, the temporal signal‐to‐noise ratio was lower, and group‐level statistical power and activation patterns of UTE maps were similar to those obtained with GE‐EPI. In addition, the UTE task‐evoked signal in CSF was negatively correlated with those in the whole cortex and in the carotid arteries and was primarily driven by the stimulus paradigm. We conclude that UTE‐fMRI can be used not only for functional studies of the human brain but also for assessing the relationship between hemodynamic and CSF signals, which can help elucidate brain homeostatic processes.

## Graphical abstract

Ultrashort and zero echo time (UTE and ZTE) MRI techniques generate functional contrast through inflow effects, making them suitable for studying cerebral blood flow and cerebrospinal fluid (CSF) dynamics. In a 7‐T study of 13 participants performing a visual task, we demonstrate that UTE produced robust, reproducible activations with group‐level performance comparable to gradient‐echo echo planar imaging (GE‐EPI). UTE revealed task‐evoked CSF signals that were anticorrelated with cortical and carotid signals, indicating its utility for assessing hemodynamic and CSF dynamics.

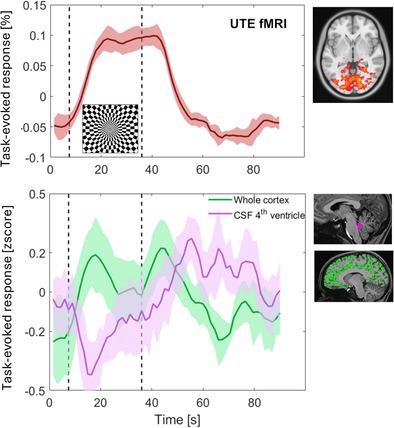

**Abbreviations**
BOLD: blood oxygenation level dependent
bpm: beat per minute
BR: breathing rate
BRV: breathing rate variability
CBF: cerebral blood flow
CSF: cerebrospinal fluid
fCNR: functional contrast‐to‐noise ratio
FD: framewise displacement
fMRI: functional magnetic resonance imaging
GE‐EPI: gradient‐echo echo planar imaging
GLM: general linear model
HR: heart rate
HRF: hemodynamic response function
HRV: heart rate variability
HWHM: half width at half maximum
LME: linear mixed effect
LMG: Lindeman, Merenda, and Gold
MNI: Montreal Neurological Institute
PP interval: pulse pulse interval
ROI: region of interest
RMSSD: root mean square of successive differences
rpm: respirations per minute
LSF: line spread function
RR interval: respiration interval
RVT: respiration volume per time
SAR: specific absorption rate
tSNR: temporal signal‐to‐noise ratio
UTE: ultrashort echo time
ZTE: zero echo time

## 1 Introduction

Functional MRI (fMRI) is one of the most widely used tools to study central nervous system activity. Standard methodologies are based on gradient‐echo echo planar imaging (GE‐EPI) to measure the blood oxygenation level dependent (BOLD) contrast [1]. Most recently, however, zero echo time (ZTE) [2] and ultrashort echo time (UTE) [3, 4, 5] acquisition techniques have been convincingly demonstrated to provide robust functional contrast (for review, see [6]) primarily of hemodynamic origin via inflow of unsaturated blood, leading to a signal that is well predicted by electrophysiological recordings [7].

With ZTE and UTE pulse sequences, data are acquired immediately after the RF excitation without waiting for the echo formation, significantly reducing signal losses and distortions which originate from susceptibility‐induced dephasing. Prior studies have demonstrated the multiple advantages of ZTE techniques in various preclinical fMRI applications, including anesthetized and awake rats [8, 9] and mice [10]. However, only pilot studies have been conducted so far in humans with ZTE‐fMRI [11, 12] or UTE‐fMRI [13, 14]. In particular, UTE‐fMRI was exploited to measure resting state in the human nasal cavity, an area that is generally precluded to standard EPI‐based fMRI given the severe signal losses originating from the magnetic susceptibility gradients at the air‐tissue interfaces [14]. Such nasal resting‐state signals were found to be strongly correlated with metrics of autonomic nervous system activity.

Because the sensitization of UTE‐fMRI contrast stems from the inflow of unsaturated spins into the imaging volume, this technique is expected to be suitable for exploring dynamics of brain neurofluids including not only cerebral blood flow (CBF), but also cerebrospinal fluid (CSF) flow. Consistent with this observation, UTE‐fMRI is able to detect a prominent resting‐state brain network that encompasses the CSF in the perivascular space around the brainstem, and with opposite polarity, the major brain vessels [14], thus putatively capturing the coupling between CSF and CBF. According to the Monroe–Kellie doctrine [15, 16, 17], global CBF and CSF dynamics are anticorrelated in order to maintain a constant intracranial pressure and thus to preserve the total intracranial volume constant.

Since neural activity is a driving factor for CSF flow along with other physiological dynamics such as respiration and cardiac cycle [18], monitoring the dynamics of CSF signals during task holds great promise for understanding brain clearance processes and their impact for supporting neuronal function and ensuring overall brain homeostasis in both physiological and pathological conditions [19]. Previous BOLD fMRI studies have focused on identifying the relationship between fMRI signals in the whole‐cortical gray matter and the fourth ventricle, used as surrogates of global CBF and CSF flow, respectively. These studies found that such signals are anticorrelated in different experimental conditions, that is, sleep and visual stimulation [15, 17]. In addition, the coupling between the global fMRI signal and CSF flow has been shown to be altered in several neurological disorders, including Alzheimer's disease‐ [20], traumatic brain injury [21], small vessel disease [22], depressive disorder [23], and Parkinsons's disease [24, 25]. While such observations are promising, BOLD fMRI is not an ideal approach for exploring the coupling between CBF and CSF signals during neuronal activity. In fact, challenges arise from confounds related to blood oxygenation and from the inherent limitations in head coverage imposed by the need of prescribing the edge of the acquisition volume at the level of the fourth ventricle to maximize CSF inflow sensitization [26]. An alternative method for the simultaneous investigation of BOLD fMRI and phase‐contrast CSF flow using EPTI [27] has recently been introduced and showed promising potential to reveal neural influences on CSF flow [28]. Alternatively, a methodology such as UTE‐fMRI is suitable, because it is directly and specifically linked to the neurofluid dynamics of interest, and because it enables wide coverage of the head and neck, which is useful for assessing neurofluid dynamics beyond the brain. In addition, previous resting‐state fMRI studies at 7 T revealed that UTE detects the intrinsic CSF network and CSF/Vessel network better than GE‐EPI [14], thus providing additional evidence corroborating the use of UTE for study of neurofluid dynamics.

Based on these premises, here we exploited UTE‐fMRI in humans for comprehensive functional studies at high magnetic field 7 T during visual stimulations. GE‐EPI data with matched nominal resolution and coverage were also acquired for qualitative comparisons. First, we demonstrate that UTE detects local brain activations in the visual cortex at both single subject and group levels. Second, we provide evidence that UTE detects robust anticorrelations of task‐evoked CSF signals with both global cortical signals and carotid‐based signals, reflective of a contrast that is directly linked to neurofluid dynamics and consistent with the Monroe–Kellie doctrine.

## 2 Methods

### 2.1 Participants

Thirteen healthy adult volunteers (age mean ± SD = 44.6 ± 17.6 years, 6 females/7 males) were recruited for the study. Exclusion criteria included age below 18 years old, incompatibility with MR safety criteria, and major neurological and psychiatric pathologies. This study was carried out in accordance with the recommendations of The Code of Federal Regulations, Institutional Review Board. Written informed consent from all participants was obtained before the study in accordance with the Declaration of Helsinki. The protocol was approved by the Institutional Review Board: Human Subjects Committee of the University of Minnesota.

### 2.2 Data Acquisition

Whole brain images were acquired on a 7‐T Siemens Magnetom scanner with a single transmit and 32‐channel receive NOVA head coil. UTE fMRI acquisitions were performed using a slab selective UTE sequence with radial center‐out readout, with a 192‐mm‐thick slab and 1070 radial views to cover a FOV = 192 × 192 × 192 mm^3^, with a final spatial resolution of isotropic 2‐mm voxel size. Other parameters were spoke repetition time (spoke‐TR) = 1.4 ms, echo time (TE) = 0.12 ms, flip angle = 2°, readout bandwidth 96,153.0 Hz, 96 readout points for each spoke, volume‐TR = 1.5 s, and 224 volumes. GE‐EPI fMRI acquisitions were performed with matched spatial coverage and resolution, and with same temporal resolutions, using a 2D GE SMS/MB EPI with 95 slices, volume‐TR = 1.5 s, TE = 22.2 ms, MB factor 4, FOV = 256 × 256, and 2‐mm isotropic voxels. Two anatomical acquisitions were also included in the protocol. The first was an MP2RAGE [29] with 240 slices, TR = 5000 ms, FOV = 240 × 225 mm, flip angle = 4°, TE = 2.27 ms, and spatial resolution 0.75 × 0.75 × 0.8 mm^3^. The second was a high‐resolution UTE sequence acquired with the following parameters: 4096 radial views, 24 radial interleaves, FOV = 192 × 192 × 192 mm^3^ with an isotropic 0.75 mm^3^ spatial resolution, TR = 3 ms, TE = 0.11 ms, and flip angle = 3.5°. B_0_ shimming was performed with standard vendor procedures and same shimming parameters were used for both UTE and GE‐EPI. The specific absorption rate (SAR) was calculated within the sequence, monitored using the vendor‐provided system, and maintained below regulatory limits. During the functional scanning, the physiological status of the participants was monitored and recorded by means of a respiratory belt and a pulse plethysmograph using Siemens PMU systems (Erlangen, Germany) with both signals sampled at 400 Hz. The recording of the physiological signals was prolonged for 2 min after the end of each scan to obtain heart rate variability (HRV) and breathing rate variability (BRV) measurement of the same size as the fMRI dataset. UTE image reconstruction was performed offline with a custom program written in C/CUDA (NVIDIA, Santa Clara, CA). K‐space sampling density, including the effects from the ramp sampling, was compensated by the iterative density correction [30]. The density‐corrected radial k‐space data were reconstructed to 3D images with nonuniform fast Fourier transform [31].

### 2.3 Visual Stimulation

The block design stimulation paradigm consisted of three repetitions of a 60 s OFF and 30 s ON blocks with an extra baseline (OFF condition) at the end. The ON stimulus consisted of a circular black and white checkerboard rotating at 15 Hz, while the OFF‐baseline condition consisted of a gray background. Stimulus presentation was controlled using Psychopy Toolbox (2022.2.5) and custom scripts. Participants were instructed to remain as still as possible and to keep looking at the center white fixation cross throughout the runs. To minimize head motion, padding was also placed around the head. To verify the level of attention throughout the experiment, participants were instructed to press a button when the fixation cross (“+”) was briefly replaced by a fixation “x” (randomly four times during baseline conditions). Responses were recorded using an MRI‐compatible button box.

### 2.4 Imaging Data Processing

#### 2.4.1 Preprocessing Steps

Task‐fMRI data were processed using FSL and SPM toolboxes and custom MATLAB and Python scripts. For the UTE task‐fMRI, the preprocessing steps included the removal of four dummy volumes and 3D rigid body motion correction, where all volumes were aligned to the first volume. For GE‐EPI data, the slice scan timing correction was performed in addition to the preprocessing steps of task‐fMRI UTE. In order to account for residual motion‐related spikes, the framewise displacement (FD) time‐series was calculated [32] and a threshold of FD > 0.5 mm was used to identify high‐motion volumes. A scrubbing procedure was applied to those volumes by removing and replacing them with linear interpolation.

UTE‐fMRI images were then aligned to respective MP2RAGE images with a two‐step procedure: First, the low‐resolution UTE (fMRI) images were aligned to the high‐resolution UTE images, then the high‐resolution UTE images were coregistered to the MP2RAGE images, both steps using FLIRT. GE‐EPI images were also coregistered to the MP2RAGE images using FLIRT. MP2RAGE images were successively processed with presurfer (https://github.com/srikash/presurfer) MATLAB scripts before spatial segmentation with SPM12. The tissue maps were then used for DARTEL normalization to the standard Montreal Neurological Institute (MNI) space and for the automatic segmentation using FreeSurfer (http://surfer.nmr.mgh.harvard.edu/). Finally, using the calculated DARTEL deformation fields, fMRI data (both UTE and GE‐EPI) were normalized to the standard MNI space while applying a spatial Gaussian smoothing of 2‐mm FWHM for the group analyses.

#### 2.4.2 Calculation of Activation Maps

To quantify the extent of stimulus‐evoked activation, a general linear model (GLM) was performed for single‐subject in both native space and MNI space (first level analysis) and for group analysis in the MNI space (2nd level analysis). For both UTE and GE‐EPI, design matrices were generated by the convolution of the SPM canonical hemodynamic response function (HRF) with a “boxcar” function representing the onset and offset of the stimuli. Additional three rotations and three translation vectors were included from the motion correction processes as confounding factors. High‐pass filter with cut‐off at 128 s was also included. For the single‐subject analysis, the significance level was set at *p* < 0.001, and only significant clusters in the occipital area were considered for further analyses. Activation extent was quantified and compared across modalities using the number of voxels in each subject's activation clusters. The dice similarity coefficient was calculated for each subject using the binarized activation clusters to quantify the activation similarity across imaging modalities. Group‐level analysis was performed using a one‐sample *t*‐test design on the contrast of interest from the beta values obtained in the single‐subject analysis. Statistical significance on the final group‐level t‐maps was assessed with a voxel‐level threshold of *p* < 0.001, and minimum cluster size threshold was estimated with Monte Carlo simulations [33] using AlphaSim implemented in DPABI [34]; the minimum cluster size was set in such a way that an average of 5% false positive clusters falling within the search volume were counted in 1000 randomly generated images to which the same threshold was applied.

#### 2.4.3 Quantification of Functional Contrast, Functional Contrast‐to‐Noise Ratio (fCNR) and Temporal Signal‐To‐Noise Ratio (tSNR)

**FIGURE 1:**
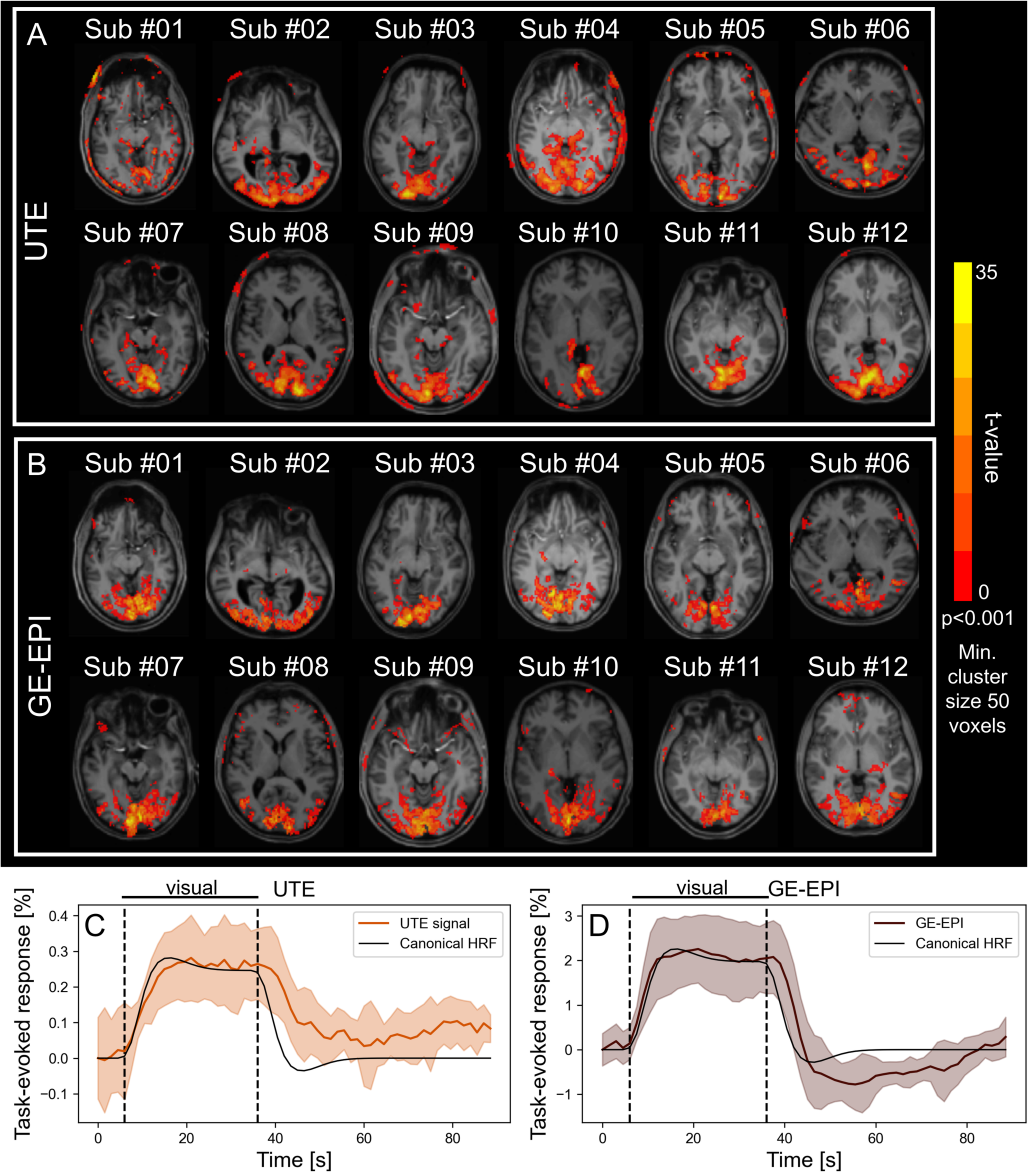
Subject‐level results of the task‐fMRI visual stimulation. Single subject t‐maps of the functional activation in the occipital cortex when fMRI data is acquired with UTE (A) and GE‐EPI (B). Task‐evoked average responses are obtained by averaging the time courses aligned to each stimulus onset within the activated clusters of each subject, and they are expressed in %‐signal change (solid line represents the mean across subjects, and shaded area represents the standard deviation across subjects) for UTE (C) and GE‐EPI (D). Canonical HRF functions, scaled to the maximum of the respective task‐evoked averages are superimposed in both UTE (C) and GE‐EPI (D) as black lines. The statistical maps are overlayed on the subjects anatomical MP2RAGE images after processing with presurfer toolbox. Statistical threshold was set to *p* < 0.001 uncorrected, and clusters whose cluster size was less than 50 voxels were excluded.

The mean time course in the significant clusters from the first level analysis was extracted for each subject in the native space without any smoothing and transformed in %‐signal change according to: S_%_(t) = (S(t) − S_b_) × 100/S_b_, where S(t) is the signal intensity at time t and S_b_ is the mean signal intensity during all baseline conditions. The S_%_(t) signals were used to calculate the stimulus‐induced signal change (ΔS) as the difference between the four highest values during each stimulation block and the preceding baseline (7). The functional contrast to noise ratio (fCNR) was then quantified as the ratio between ΔS and the standard deviation of baseline temporal fluctuations. Moreover, to assess the stability of the functional signals independently from the task, the temporal signal‐to‐noise ratio (tSNR) was calculated as the ratio between the mean baseline signal and its standard deviation. Task‐evoked responses were averaged across subjects and superimposed on the standard HRF convolved with the boxcar predictor for visualization purposes (Figure 1), and correlations across measured and predicted signals were computed for quantifying the fidelity of the model. Summary statistics of the fCNR, tSNR, and the activation extent are reported as mean ± SD.

#### 2.4.4 Line Spread Function Evaluation

Image blurring was quantified in one representative participant by estimating the half width at half maximum (HWHM) of the line spread function (LSF) obtained from one‐dimensional intensity profiles for UTE‐fMRI, UTE‐anatomical, and GE‐EPI. In particular, for each image modality, intensity values were sampled along a line encompassing a sharp edge, that is, white matter‐lateral ventricle. The resulting profiles were modeled using a Gaussian function with a nonlinear least‐squares fitting, and the obtained Gaussian standard deviation (σ) was used to analytically calculate the $\text{HWHM}=\sqrt{2\ln2}\sigma$. The image blurring introduced by the spatial smoothing commonly applied in group level fMRI studies (Gaussian smoothing, FWHM = 2 mm and 6 mm) was also evaluated. For visualization purposes, we finally calculated the group fMRI maps from UTE and GE‐EPI data after they were smoothed with FWHM levels found to led to similar HWHM (i.e., similar effective resolutions), namely, 0 mm (i.e., no smoothing) for UTE‐fMRI and 6 mm for GE‐EPI.

#### 2.4.5 Regions of Interest (ROIs)

Three ROIs were defined in each subject. In particular, cortical gray matter and the fourth ventricle ROIs were defined from the automatic segmentation generated by FreeSurfer and eroded by one voxel to minimize partial volume effects. One ROI encompassing both right and left internal carotid arteries was used to obtain a global inflow proxy (rather than a direct measure of posterior circulation). Such ROI was semi‐automatically obtained by setting an intensity threshold within the areas surrounding each carotid artery using MP2RAGE images and then manually adjusted to exclude other tissue types. In addition, the mask of statistically significant clusters from the group‐level analysis was included after registering it to individual subjects using the inverse DARTEL transformations.

#### 2.4.6 Correlations of Task‐Evoked Dynamics Between ROIs

The average time‐series from all the voxels in each ROI was obtained from each UTE fMRI dataset. The task‐evoked response was calculated by averaging time courses locked to each stimulus onset. Pearson's correlation coefficient was calculated between the task‐evoked average signals of the ROIs. The same analysis was only performed on UTE data because the GE‐EPI acquisition was not optimized to enhance flow‐related signals. All the ROI signals were extracted using the nonwarped and nonsmoothed data to minimize contamination of the signal from different tissues.

#### 2.4.7 Physiological Data Processing

For processing the physiological signals, we used PhysioNet Cardiovascular Signal Toolbox [35] implemented in MATLAB, python‐based scripts to create fMRI predictors with physiological signals provided by BrainVoyager (https://github.com/BrainInnovation/physiocorr) and custom scripts. Pulse signals were imported in MATLAB, synchronized with the MRI acquisition and then smoothed with a Savitzky–Golay FIR smoothing filter (order 3, frame size 67 samples). Signals from the respiratory belt were also imported in MATLAB and synchronized with the MRI acquisition. Pulse and respiration wave onsets were detected by analyzing the slope sum function, and the time between successive pulse and respiration onsets (PP and RR intervals, respectively) was calculated. The PP and RR intervals were used to obtain HRV and BRV time series. Particularly, the root mean square of successive differences (RMSSD) in PP or RR intervals was calculated in adjacent time windows of 100 s with a timestep of 1 s [36]. From the pulse and respiration signals, also the heart rate (HR) and the breathing rate (BR) were calculated as time difference between successive maximum peaks expressed in beat per minute (bpm) and respirations per minute (rpm), respectively. Finally, the respiration volume per time (RVT), which is a measure proportional to the BR and depth of respiratory signal at each time point, was calculated according to [37] as the product between breathing depth and rate. All the obtained measures were resampled at the fMRI temporal resolution (1.5 s). To quantify the contribution of physiological activity to the fMRI signal temporal evolution at group level, a linear mixed model was performed in MATLAB along with a relative importance computation. Specifically, the fMRI signals from each ROI were used as dependent variables, while the five physiological metrics (HR, BR, HRV, BRV, and RVT) plus the predictor of the visual task (boxcar signal convolved with the HRF) were used as independent variables. The subjects were accounted as a random effect variable. Results were considered significant after Bonferroni correction for multiple comparisons on the number of independent tests (*n* = 3 independent ROIs). We then used the Lindeman, Merenda, and Gold (LMG) metric [38] to decompose the total explained variance in the model into multiple nonnegative contributions from the individual fixed effect (LME) predictors.

## 3 Results

All participants completed the experimental sessions. The SAR of the UTE acquisitions remained well below the regulatory safety limits, namely, ~10% during UTE‐fMRI and ~30% during UTE anatomical acquisitions. One participant's data were excluded from the analysis due to technical problems which caused the acquisition of a shorter fMRI time series; therefore, all the analyses were performed on 12 participants (age mean ± SD = 42.7 ± 16.9 years, 6 females/6 males). The scrubbing procedure resulted in the removal of 7 volumes from 3 participants in the UTE dataset and 14 volumes from one participant in the GE‐EPI dataset.

### 3.1 Task‐Evoked Brain Responses

**FIGURE 2:**
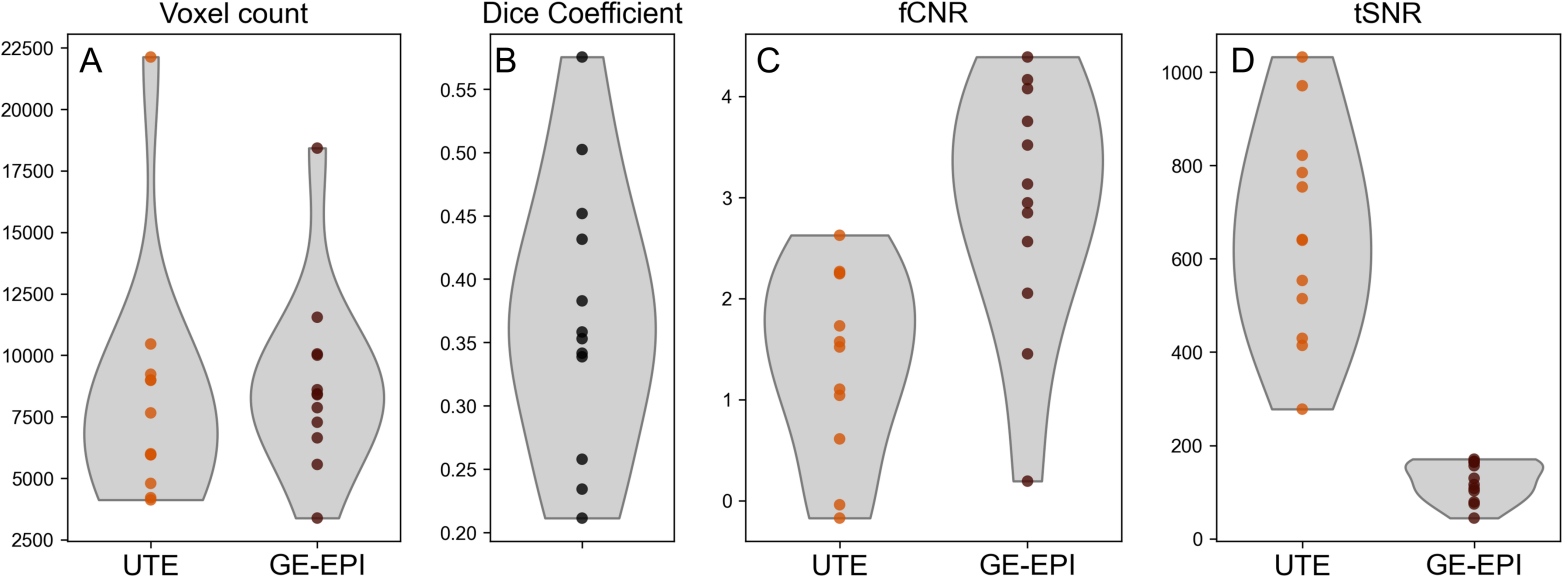
Features of subject‐level fMRI data. (A) Violin plots and individual scatter plots of the activation extent expressed as number of voxels with both imaging modalities. (B) Violin plot and individual scatter plots of the dice coefficient calculated for each subject between the activation clusters in UTE and GE‐EPI respectively. (C) Violin plots and individual scatter plots of the fCNR with both imaging modalities. (D) Violin plots and individual scatter plots of the tSNR calculated from the time‐courses of last baseline of the task function data with both imaging modalities.

At the single‐subject level, the task‐fMRI results reveal a stimulus‐evoked functional activation in the primary visual cortex in all subjects for both UTE (Figure 1A) and GE‐EPI (Figure 1B) acquisitions. The functional contrast expressed as task‐evoked %‐signal change (averaged across subjects) in the activated clusters was lower for UTE (~0.2%, Figure 1C) than for GE‐EPI (~2%, Figure 1D). The fidelity of the model using the canonical HRF was similar across modalities, namely, Pearson's *r* = 0.90 for UTE and *r* = 0.95 for GE‐EPI. The activation extent, expressed as the number of voxels in the significant clusters, was 8850 ± 3706 voxels for GE‐EPI and 8208 ± 4868 voxels for UTE‐fMRI (Figure 2A), and the spatial similarity as expressed by the dice coefficients was 0.37 ± 0.10 (Figure 2B). fCNR of GE‐EPI and UTE‐fMRI data were 2.9 ± 1.2 and 1.40 ± 0.9, respectively (Figure 2C). On the other hand, independently from the task‐related activation, tSNR of UTE‐fMRI was 653 ± 229, while for GE‐EPI it was 123 ± 43 (Figure 2D).

**FIGURE 3:**
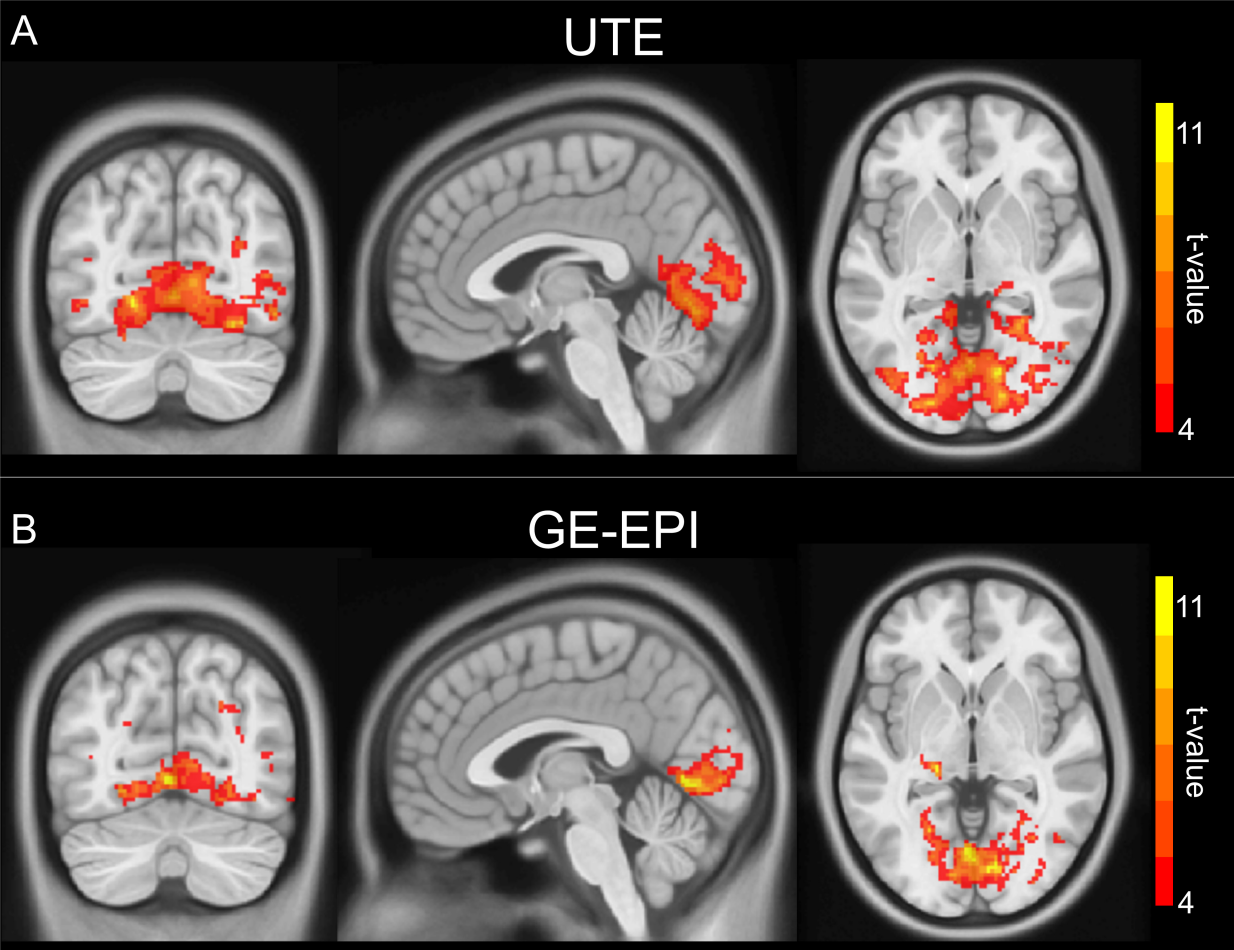
Group‐level results of task‐fMRI during visual stimulation. (A) Group‐level t‐map of the functional activation in the occipital cortex when fMRI data is acquired with UTE. (B) Group‐level t‐map of the functional activation in the occipital cortex when fMRI data is acquired with GE‐EPI. T‐maps were overlayed on the MNI anatomical template. Statistical threshold was set to *p* < 0.001 uncorrected; setting of minimal cluster size is described in methods.

Consistent patterns of activation across modalities were observed also at group level (Figure 3A,B), with peak *t*‐scores occurring in the lingual gyrus (UTE MNI coordinates −22, −54, −10; *t*‐value = 16.3, *p* < 0.001; GE‐EPI MNI coordinates −2, −68, 0; *t*‐value = 14.5, *p* < 0.001) and comparable average *t*‐score in the clusters of activation (5.5 ± 1.3 in UTE and 5.2 ± 1.2 in GE‐EPI).

### 3.2 LSF Evaluation

Figure S1 represents the evaluation results of image blurring, quantified by the HWHM of the line spread function. As compared to high resolution UTE acquisitions (HWHM = 1.87 mm, Figure S1A), the undersampling in UTE‐fMRI results in increased spatial blurring (HWHM = 4.32 mm, Figure S1B). UTE‐fMRI also exhibits higher spatial blurring than GE‐EPI, specifically HWHM = 4.32 mm vs. 2.36 mm (Figure S1B,C, respectively). When spatial smoothing is applied, like done in group level fMRI studies, the LSF of UTE‐fMRI becomes more similar to that of GE‐EPI (HWHM = 4.54 mm vs. 2.87 mm with 2‐mm Gaussian smoothing, Figure S1D,E, respectively; and HWHM = 5.13 mm vs. 4.38 mm with 6‐mm Gaussian smoothing, Figure S1F,G, respectively), as highlighted by the line profiles encompassing the sharp edge at the interface of white matter and the left ventricle (Figure S1H). The group fMRI maps, obtained from UTE and GE‐EPI data after applying FWHM smoothing levels that led to similar HWHM (namely, 0 mm for UTE and 6 mm for GE‐EPI), are shown for visualization purposes in Figure S2A,B, respectively. In UTE, peak *t*‐score occurred in the fusiform gyrus (coordinates 24, −58, −12) with *t*‐value = 14.9, *p* < 0.001; in GE‐EPI, peak *t*‐score occurred in the lingual gyrus (MNI coordinates −2, −76, −4) with *t*‐value = 14.6, *p* < 0.001. Average *t*‐scores in the clusters of activation were 5.4 ± 1.2 in UTE and 6.0 ± 1.4 in GE‐EPI, overall similar to what reported in Figure 3.

### 3.3 Signal Dynamics in Activated Occipital Cortex, Whole Brain, CSF, and Carotids

**FIGURE 4:**
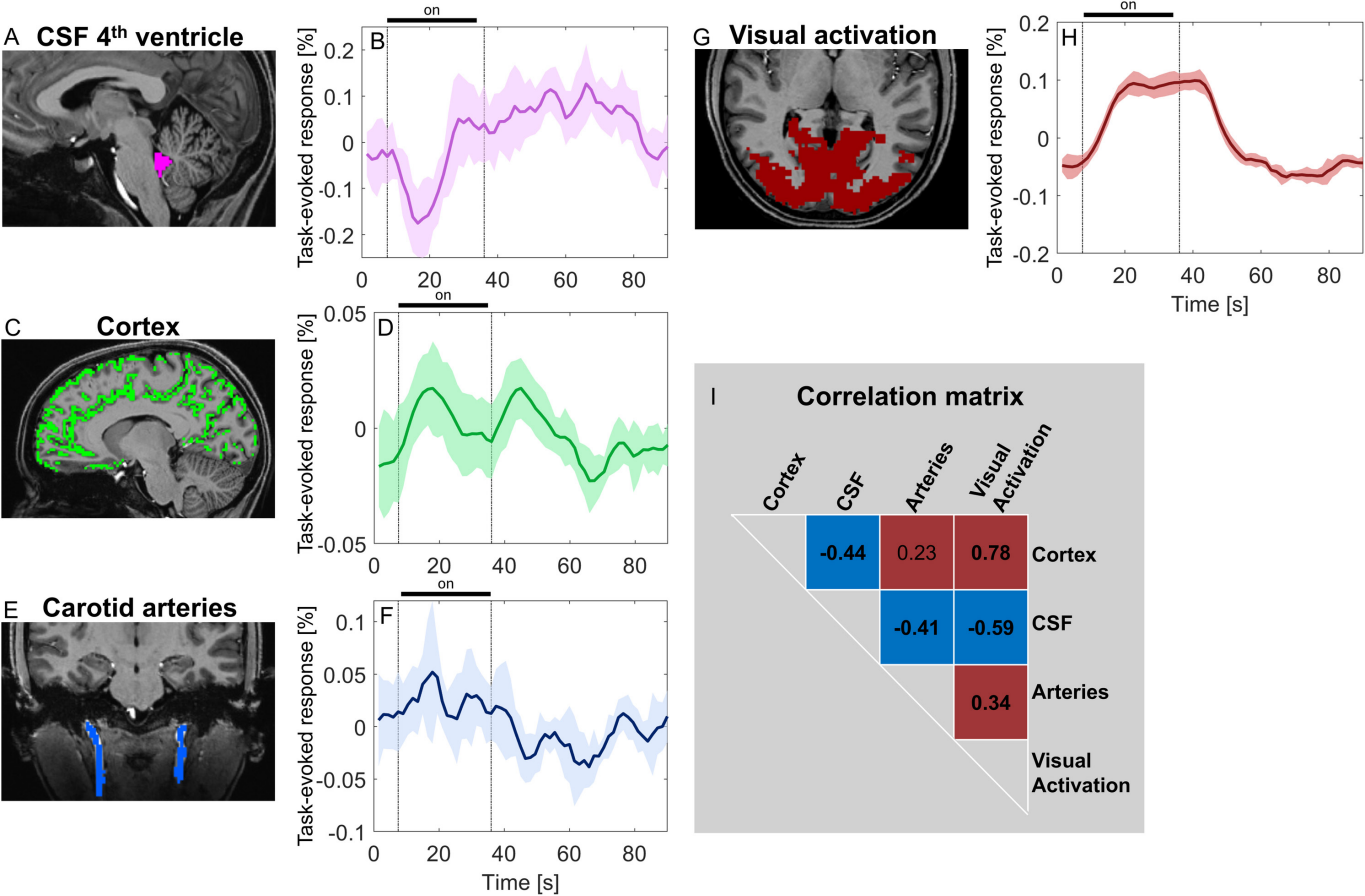
Task‐evoked responses and their correlations across ROIs. ROI locations and ROI signals are shown in (A, B) for CSF, in (C, D) for whole cortex, in (E, F) for carotid arteries, and in (G, H) for visual activation cluster. Time courses are shown after applying a 6‐s moving window smoothing. Solid lines represent the average signals across (*n* = 12) subjects, while shadows represent the standard error across subjects. Dotted vertical lines indicate the visual task on and off. (I) The table shows Pearson's correlation coefficients between each pair of ROIs, where blue and red backgrounds indicate negative and positive correlations, respectively, and statistically significant correlations are highlighted in bold.

We then focused on the functional signals obtained in the whole cortex, in the fourth ventricle, in carotids, and in the activated area in occipital cortex. As shown in Figure 4, the average task‐evoked CSF signal exhibits a clear decrease at the beginning of the visual stimulation followed by an increase (Figure 4A,B), while an opposite behavior is observed for the CBF‐mediated cortical signal (Figure 4C,D), with a significant negative correlation between the two signals (*r* = −0.44, *p* = 0.0004). Similar behavior is observed for the UTE signal within the internal carotid arteries (Figure 4E,F), leading to a significant negative correlation between the signals in CSF and internal carotid arteries (*r* = −0.41, *p* = 0.001). Moreover, the signal in the cluster of visual activation (Figure 4G,H) was positively correlated with the global signal in the whole cortex (*r* = 0.78, *p* < 0.0001) and in the carotid arteries (*r* = 0.34, *p* = 0.006) and negatively correlated with the CSF signal (*r* = −0.59, *p* < 0.0001). In addition, a positive correlation (*r* = 0.23, *p* = 0.07), albeit not statistically significant, occurred between UTE signals in the whole cortex and in the carotids (see Figure 4I for the complete correlation matrix).

### 3.4 Influence of Physiological Parameters

**FIGURE 5:**
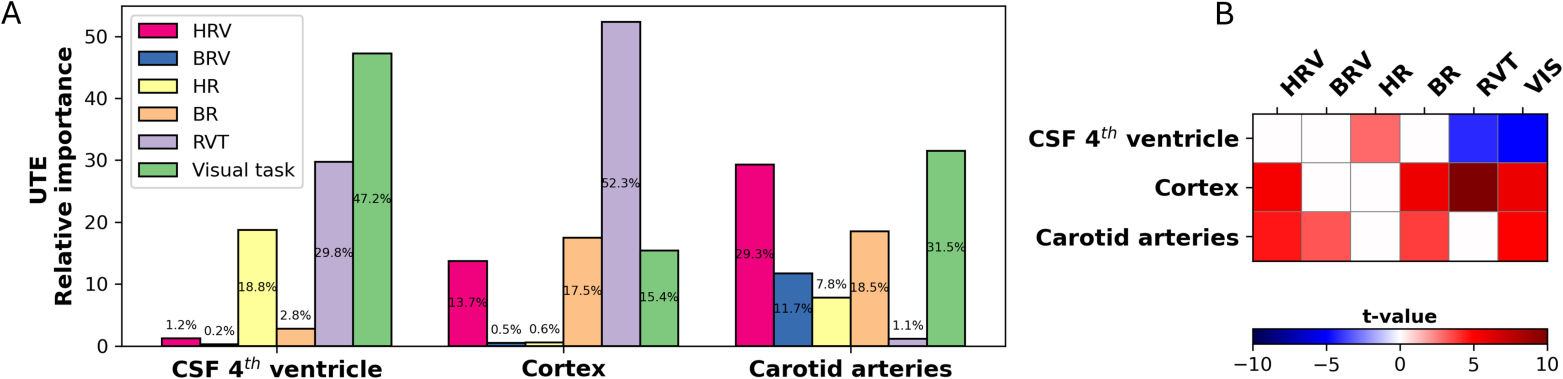
Results of the linear mixed effect (LME) model with relative importance computation of the physiological parameters and visual task. (A) Percentage of relative importance of the predictors (identified by different colors as described in the legend) for the three ROIs (CSF in the fourth ventricle, cortex, and carotid arteries). (B) Summary matrix of the LME results. Redish and bluish shaded cells indicate, respectively, positive and negative t‐values that are considered significant. Nonsignificant effects are indicated with white cells. BR, breathing rate; BRV, breathing rate variability; HR, heart rate; HRV, heart rate variability; RVT, respiration volume per time.

Only data from 9 out of 12 participants were considered for the analysis of physiological data due to technical problems during the physiological recordings. The importance analysis showed that the highest importance in the CSF variability is attributed to the visual task followed by the RVT (Figure 5A), both of which are significantly anticorrelated with the CSF fMRI signal (Figure 5B). On the other hand, the highest importance in the cortical‐GM signal variability is attributed predominately to the RVT followed by the BR, both significantly positively correlated with the fMRI signal in the whole cortex. The relative importance appears more distributed when considering the signal in the carotid arteries, with highest contributions from the visual task and the HRV.

## 4 Discussion

In the current study, we demonstrated the feasibility of detecting task‐evoked functional contrast in humans by using MRI sequences with ultra‐short echo time. The visual task experiment demonstrated robust UTE‐fMRI responses in the primary visual cortex in the occipital lobe, spatially similar to those obtained with GE‐EPI at single subject and group levels. While previous work has convincingly demonstrated the robustness of ZTE techniques in preclinical applications, only a few previous studies so far had attempted task‐based fMRI with UTE or ZTE in humans. In particular, Kim and colleagues detected negative responses during a visual task with UTE [13], while Özen and colleagues reported preliminary findings of responses consistent with the results obtained in this study [12]. Therefore, our results confirm that brain activation maps can be obtained in humans even in the absence of an echo, consistent with extensive evidence previously reported in rodent studies [7, 8, 9, 39, 40, 41, 42, 43, 44, 45]. Moreover, while UTE‐fMRI exhibits lower fCNR than GE‐EPI, both techniques demonstrated comparable sensitivity for detecting group level task‐related activations, though at different effective spatial resolutions (Figure 3). Furthermore, UTE‐fMRI exhibited higher tSNR than GE‐EPI, which likely relates to its insensitivity to respiration‐induced dynamic field inhomogeneity [41], a major source of time course noise in EPI [46]. It should also be noted that fCNR in UTE‐fMRI may improve via further optimization of imaging parameters and reconstruction. These characteristics make UTE a valuable fMRI alternative to conventional BOLD‐fMRI, particularly at high and ultra‐high magnetic field strengths, where achieving good data quality with GE‐EPI is often challenged by field inhomogeneities that may not fully resolve even after shimming procedures. On the other hand, the high radial undersampling, which was required to achieve fMRI‐compatible temporal resolution with UTE acquisition, introduced inherent spatial blurring in the functional images. However, the image blurring, as quantified by the HWHM of the line spread function at the edge of ventricles, appeared comparable to that of GE‐EPI when applying Gaussian smoothing levels typical for group fMRI analyses (Figure S1).

As discussed above, current evidence indicates that in FID‐based sequences, such as UTE, functional contrast primarily arises from the inflow of unsaturated blood during RF excitation, leading to increased blood magnetization with rising CBF and CBV [6]. Furthermore, rodent studies have demonstrated a dependence on flip angle, consistent with a T1‐mediated mechanism [8]. Recent advancements have also shown the sensitivity of the UTE sequence to detect CBV‐ [47] and T1‐mediated [48] functional contrast when contrast agents are used. Additional T1 contributions may arise from changes in molecular oxygen [44], while other proposed mechanisms, including neuronal or glial swelling [49] and changes in arterial pressure [50], could also contribute, although their relative importance has yet to be established.

Our results also showed clear increases in task‐evoked UTE signals in the whole‐cortex and carotid arteries, mirrored by task‐evoked signal decreases in the fourth ventricle of the CSF regions. This observation was in line with recent evidence of neural‐driven brain clearance [51, 52]. Moreover, the relative importance analysis showed that the variance of the UTE CSF signal was predominantly explained by the visual task, but strong contributions were also attributed to RVT and HR. These findings collectively support the hypothesis that a sufficiently large change in blood volume, like the one driven by an intense visual stimulation, can prompt waves of CSF flow, therefore confirming that neural activity is a major modulator of the CSF flow along with the physiological drives such as cardiac and respiratory rhythms. Moreover, RVT is a measure that has been linked to arterial CO_2_ levels [53]. Induced hypercapnia (increase in CO_2_) is related to reduction of cranial CSF volume [54], and CO_2_‐induced changes in CBV have been correlated to changes of CSF inflow signal around the fourth ventricle, thus supporting a link between CSF and CO_2_ change throughout CBV changes [55, 56]. Moreover, the variance of the whole cortex UTE signal is explained to a greater extent by RVT than by the visual task, again consistent with global CBF being strongly influenced by CO_2_ fluctuations. Finally, the UTE fMRI signal in the carotid arteries appears to be modulated by multiple factors, including task‐evoked responses, autonomic activity (e.g., HRV and BRV), and respiratory dynamics. This observation is physiologically plausible, as the carotid arteries are the primary conduits for cerebral blood supply and therefore respond directly to fluctuations in metabolic demand during neural activation [57, 58]. In addition, their tone and flow are under strong autonomic regulation, with both sympathetic and parasympathetic inputs modulating vascular resistance and compliance [59]. Consequently, variations in task‐related activity, autonomic responses, and respiration can jointly shape carotid hemodynamics, thereby contributing to the observed fMRI signal.

The sensitivity of UTE with a whole‐brain slab selection to functional hyperemia relates to the inflow of unsaturated blood from arteries outside the excited slab [8], and it thus depends on the arterial transit time during which the longitudinal magnetization of the inflowing spins undergoes continuous partial saturation. The contrast disappears once inflowing spins reach steady state; when, along the flow pathway, the steady state is reached, it depends on flip angle, spoke TR, and intrinsic blood T1 [60]. While arterial spins reach the brain with a transit time of around ~1.5 s [61, 62], they also reach the CSF through the highly perfused choroid plexus, which has an arterial transit time on the order of ~1.2 s in the lateral ventricles [63] and likely even shorter in the fourth ventricle. Therefore, both CBF and CSF sensitizations appear feasible with similar sequence parameters, consistent with the results of our study. For instance, considering that blood T1 is around 2.12 s at 7 T [64], in the presence of high flip angle and short spoke‐TR of 6° and 1 ms, respectively, no contrast is expected because steady state would be reached rapidly within 1 s, which is shorter than both transit times. On the other hand, with flip angle = 2° and spoke‐TR = 1.4 ms, as used in the current study, steady state is reached in about 5 s, well beyond both arterial transit times. However, fully characterizing the sensitivity of slab‐selective UTE to CSF dynamics along the CSF circulatory pathway will require additional research, taking into account the rate of production, different velocities, and exchange rates between blood and CSF.

Recently, other MRI‐based methods have been introduced to characterize CSF dynamics, such as the use of a dedicated MRI sequence employing steady‐state free precession tagging to visualize CSF flow [65] or techniques that detect CSF inflow effects through interslice signal suppression caused by CSF inflow [66]. However, these approaches do not allow for simultaneous acquisition of neural activity and necessitate dedicated sequences and processing strategies. A recently developed alternative is phase‐contrast MRI, which has shown promise for quantifying CSF flow while simultaneously acquiring BOLD‐fMRI data [28]. Although this approach offers exciting opportunities to study the coupling between neural activity and CSF dynamics, it does not permit the direct estimation of CBF due to the confounding effects of blood oxygenation inherent to the BOLD signal. The proposed method, on the other hand, can characterize both hemodynamic and CSF responses within the same protocol, enabling straightforward signal correlation analyses that are indicative of potential couplings between neurofluid dynamics.

Finally, ROI‐averaged signals across the whole cortex revealed a temporal profile with UTE that differs from that typically reported with GE‐EPI BOLD. Specifically, the UTE signal from the entire cortex showed two distinct peaks, one during and one right after the end of the prolonged stimulation. This is an interesting observation that may reflect the different neurovascular contributions captured by this sequence, which is expected to be more sensitive to CBF and CBV changes than conventional GE‐EPI BOLD. However, more studies are warranted to elucidate its physiological origin.

Our study has several limitations that will need to be addressed in future research. The results demonstrated the ability to detect functional contrast with UTE in humans. However, further studies are needed not only to optimize the acquisition strategy in terms of spatial and temporal resolution but also to verify the generalizability of the approach to other experimental conditions, including the use of 3‐T clinical scanners and body rather than local transmit coils. Moreover, although the presented evaluations of functional sensitivity were useful to substantiate the feasibility of UTE‐fMRI in humans, they were not exhaustive. Direct comparisons of functional sensitivity between imaging methodologies are inherently challenging, in particular across techniques with different image contrasts, sampling schemes, noise characteristics, and effective resolutions, thus requiring dedicated efforts beyond the scope of the current study. In addition, due to differences in image contrast compared to standard GE‐EPI images, ad‐hoc processing pipelines, including, for example, motion correction and image registration, need to be optimized for UTE‐fMRI. Furthermore, although the canonical HRF was a reasonable choice to model the data (Figure 1), the specific HRF for UTE‐fMRI should be determined with dedicated experiments in future studies. Yet, assuming the same canonical HRF for both modalities led to only slightly inferior fidelity outcomes with UTE than GE‐EPI, consistent with the prominent hemodynamic origin of UTE‐fMRI signals in activated areas. In addition, the GE‐EPI acquisition used in this study was matched in terms of spatial coverage to the UTE acquisition and was thus not optimized to enhance CSF inflow through the first slice of the acquisition volume as it was done previously [15, 17]. Therefore, a proper comparison between the two techniques to evaluate the coupling between neurofluid signals is currently lacking. Finally, while the proposed method allows obtaining valuable information on neurofluid dynamics and their couplings, the quantification of CBF and CSF flow in physical units from UTE signals is still an unfulfilled goal. Systematic evaluations of the trade‐offs between sequence parameters, further investigations into the sensitivity of contrast to CSF dynamics, as well as comparisons with complementary techniques such as phase‐contrast MRI [27] or ultra‐long‐TE arterial spin labeling [67], will be important directions for future work.

In conclusion, we demonstrated that task‐based functional activation in the human brain can be achieved using UTE sequences at 7 T with a brain transmit/receive coil. Furthermore, UTE‐fMRI proved to be a valuable tool for measuring the coupling of task‐evoked signals in the whole cortex and the CSF, an opportunity that may contribute to a better understanding of global brain clearance processes and how the brain meets local demands by rearranging global resources during different brain states.

## Author Contributions

Conceptualization: S.P., S.Mi., S.Ma. Data curation: S.P., S.Ma. Formal analysis: S.P., S.Ma. Investigation: S.P., E.P., P.F., D.L.R., J.S.V., O.G., S.Mi., S.Ma. Methodology: S.P., S.Mi., S.Ma. Software: S.P. Visualization: S.P. Writing – original: S.P., S.Ma. Writing – review and editing: S.P., E.P., P.F., D.L.R., J.S.V., O.G., S.Mi., S.Ma.

## Funding

This work received support from the National Institutes of Health: P41 EB027061. The content is solely the responsibility of the authors and does not necessarily represent the official views of the funding agencies.

## Conflicts of Interest

The authors declare no conflicts of interest.

## Supporting information


**Figure S1:** Blurring estimation in images acquired with different sequences in one representative participant. (A) UTE acquired at high resolution (UTE‐HR); (B, C) UTE acquired at fMRI resolution (UTE‐fMRI) and spatial‐resolution matched GE‐EPI with no spatial smoothing applied during fMRI processing, (D, E) UTE‐fMRI and GE‐EPI with Gaussian spatial smoothing at FWHM = 2 mm applied during fMRI processing, (F, G) UTE‐fMRI and GE‐EPI with Gaussian spatial smoothing at FWHM = 6 mm applied during fMRI processing. Panel (H) shows the line profile encompassing a sharp edge (red dotted lines on the images) for each image and the corresponding Gaussian fit used to estimate half width at half maximum (HWHM). sm: extent, expressed in mm, of the FWHM of the Gaussian spatial smoothing applied during fMRI processing.
**Figure S2:** Group‐level fMRI maps during visual stimulation obtained from unsmoothed UTE data (A) and 6‐mm smoothed GE‐EPI data (B). T‐maps were overlayed on the MNI anatomical template. Statistical threshold was set to p < 0.001 uncorrected; setting of minimal cluster size is described in methods.

## Data Availability

Unprocessed data are not publicly available due to the sensitive nature of the dataset. However, data will be provided at a reasonable request addressed to the corresponding author.
