# Supplementary material for: Task‐Evoked Functional Activation and Coupling With CSF Flow Detected in the Human Brain With Ultrashort Echo Time fMRI at 7 T
Source: NMR Biomed. 2026 Jun 21;39(8):e70342. doi: 10.1002/nbm.70342 (PMC13284447; doi:10.1002/nbm.70342)
Supplement: Supplementary file 1 — Figure S1: Blurring estimation in images acquired with different sequences in one representative participant. (A) UTE acquired at high resolution (UTE‐HR); (B, C) UTE acquired at fMRI resolution (UTE‐fMRI) and spatial‐resolution matched GE‐EPI with no spatial smoothing applied during fMRI processing, (D, E) UTE‐fMRI and GE‐EPI with Gaussian spatial smoothing at FWHM = 2 mm applied during fMRI processing, (F, G) UTE‐fMRI and GE‐EPI with Gaussian spatial smoothing at FWHM = 6 mm applied during fMRI processing. Panel (H) shows the line profile encompassing a sharp edge (red dotted lines on the images) for each image and the corresponding Gaussian fit used to estimate half width at half maximum (HWHM). sm: extent, expressed in mm, of the FWHM of the Gaussian spatial smoothing applied during fMRI processing. Figure S2: Group‐level fMRI maps during visual stimulation obtained from unsmoothed UTE data (A) and 6‐mm smoothed GE‐EPI data (B). T‐maps were overlayed on the MNI anatomical template. Statistical threshold was set to p < 0.001 uncorrected; setting of minimal cluster size is described in methods. [file NBM-39-e70342-s001.docx]

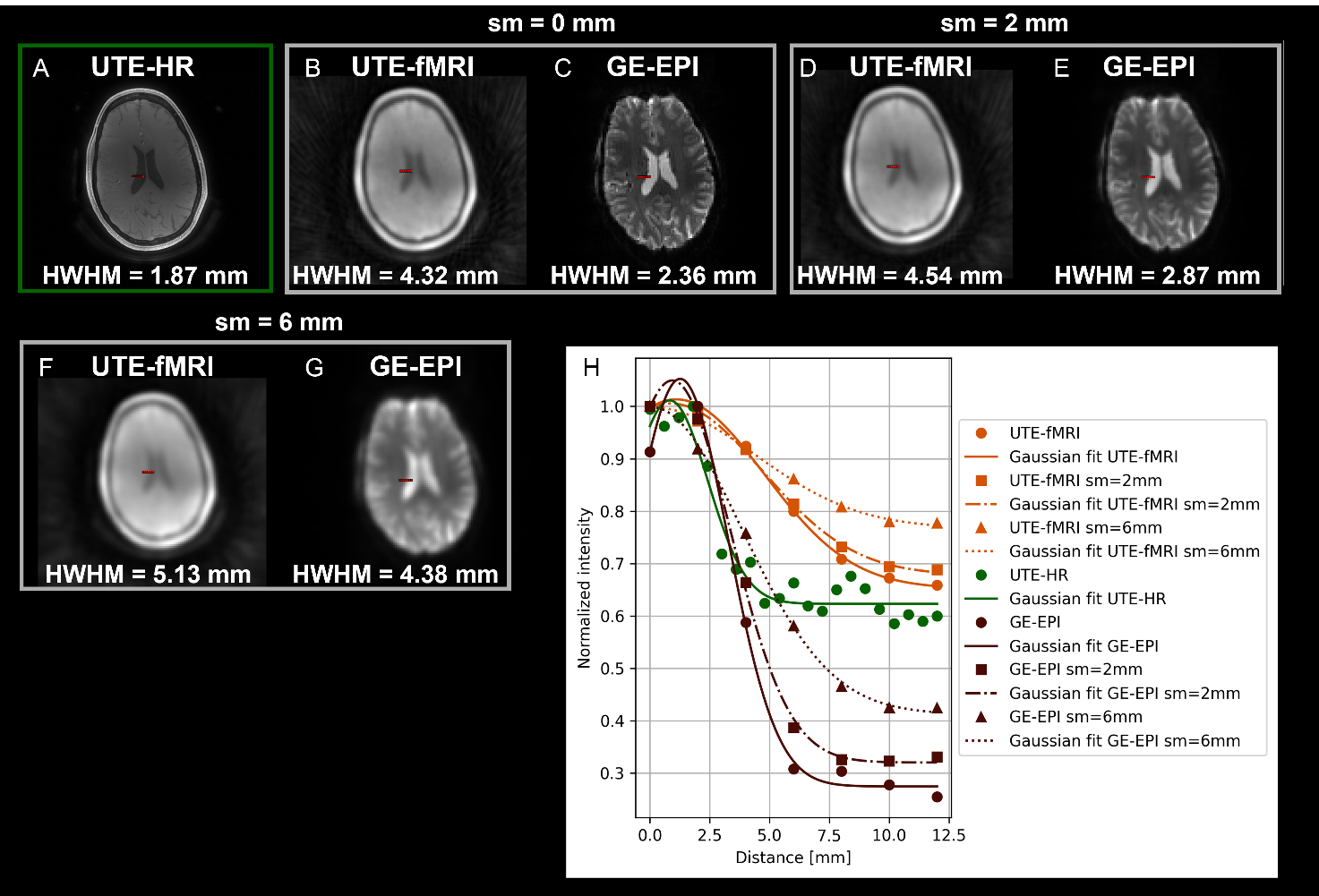


**Figure S1.** **Blurring estimation in images acquired with different sequences in one representative participant.** (A) UTE acquired at high resolution (UTE-HR); (B, C) UTE acquired at fMRI resolution (UTE-fMRI) and spatial-resolution matched GE-EPI with no spatial smoothing applied during fMRI processing, (D, E) UTE-fMRI and GE-EPI with Gaussian spatial smoothing at FWHM = 2 mm applied during fMRI processing, (F, G) UTE-fMRI and GE-EPI with Gaussian spatial smoothing at FWHM = 6 mm applied during fMRI processing. Panel (H) shows the line profile encompassing a sharp edge (red dotted lines on the images) for each image and the corresponding Gaussian fit used to estimate half width at half maximum (HWHM). sm: extent, expressed in mm, of the FWHM of the Gaussian spatial smoothing applied during fMRI processing.


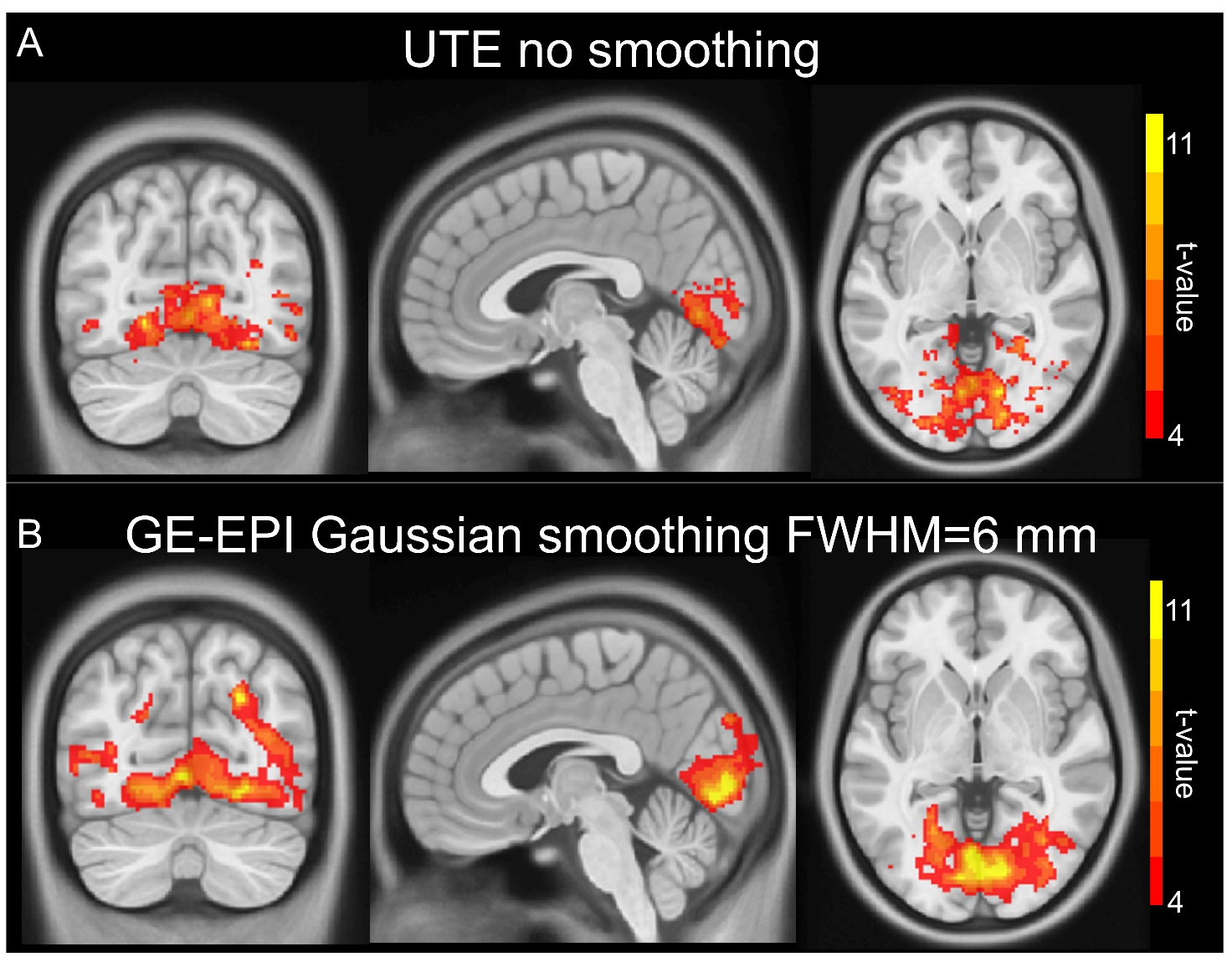


**Figure S2.** Group-level fMRI maps during visual stimulation obtained from unsmoothed UTE data (A) and 6-mm smoothed GE-EPI data (B). T-maps were overlayed on the MNI anatomical template. Statistical threshold was set to p<0.001 uncorrected; setting of minimal cluster size is described in methods.
